# A comparative study between sacral neuromodulation and intravesical botulinum toxin injection for patients with refractory overactive bladder

**DOI:** 10.1080/2090598X.2020.1740391

**Published:** 2020-03-23

**Authors:** Issam S. Al-Azzawi, Haider T. Al-Hindawi

**Affiliations:** a Department of Urology, Al-Mustansiriya University, Baghdad, Iraq; b Department of Urology, Al-Yarmouk Teaching Hospital, Baghdad, Iraq

**Keywords:** Refractory overactive bladder, botulinum toxin, sacral neuromodulation, treatment response, quality-of-life score

## Abstract

**Objective:**

To compare the efficacy, safety, patient compliance and quality of life (QoL) (early and at 6 months after treatment), in a group of Iraqi female patients with refractory overactive bladder (OAB), treated with intradetrusor botulinum toxin A (BTX) injections vs sacral neuromodulation (SNM).

**Patients and methods:**

A prospective, clinical interventional study of 37 female patients assessed by history, physical examination, voiding diary, ultrasonography (US), and urodynamics. The patients were assigned to one of two groups: Group 1, treated with cystoscopic BTX injections; and Group 2, treated with SNM. Response to treatment was assessed by voiding diary, the Treatment Benefit Scale, a modified Quality of Life scale, urine culture, and abdominal US.

**Results:**

The mean age of the patients in Group 1 (BTX) was 43.8 years and in Group 2 (SNM) was 37.2 years. OAB-wet was diagnosed in 11 patients in Group 1 and 10 in Group 2. At the 6-month follow-up there were 14/16 and 12/15 positive responders, in groups 1 and 2, respectively; with no major complications. All the responders had a significant improvement in their overall QoL after both types of treatment.

**Conclusions:**

Both BTX and SNM, in our experience, were safe and effective in managing our patients with refractory OAB after 6 months of follow-up, which was also reflected by an improvement in their QoL.

**Abbreviations:**

BTX: botulinum toxin A; IPG: implantable pulse generator; OAB: overactive bladder; PVR: post-void residual urine; QoL: quality of life; SNM: sacral neuromodulation; UDS, urodynamics; UI, urinary incontinence

## Introduction

Treatment of patients with overactive bladder (OAB) may require different lines and approaches; starting with lifestyle modifications, pelvic ﬂoor muscle training, bladder re-training and medications (antimuscarinics and β-agonists).When these therapies fail or are intolerable for patients, other options are currently available, e.g. sacral neuromodulation (SNM), percutaneous tibial nerve stimulation and botulinum toxin A (BTX) injections, which should be considered before more invasive options, such as bladder augmentation or urinary diversion [1–4]

In fact, OAB is not a life-threatening condition, but it can have a profound effect on quality of life (QoL), which is why in choosing a treatment its potential benefit must be weighed against its risk of adverse events. Patients with OAB are significantly burdened by their symptoms, and further burdened when seeking treatment relief of their symptoms that can be costly and time consuming. These burdens significantly lower QoL scores in such patients and for many it leaves them socially isolated and psychologically disturbed [5,6].

Treatment success is usually judged by improvement in voiding diaries, subjective patient satisfaction, and QoL scores [7].

In Iraq, OAB is underestimated, as many patients do not consult doctors because of embarrassment, and some patients even believe that these symptoms are natural and due to ageing. Until recently, the unavailability of second- or third-line treatment options in Iraq was an important factor contributing to frustration of both patients and their doctors.

After 2010, some Iraqi urologists started to use intradetrusor BTX injections and in 2015 Al-Azzawi and Al-Tamimi [7], introduced sacral neuromodulation (SNM) for the first time in Iraq, for the treatment of various lower urinary tract dysfunctions, including patients with refractory OAB.

The aim of the present study was to compare the effectiveness, safety, patient compliance, patient QoL, and cost effectiveness of intradetrusor BTX injections vs SNM, in a group of Iraqi patients with refractory idiopathic OAB. To our knowledge, this is the first study that compares the outcome of these two recently introduced treatment options in Iraq and their impact on the QoL of Iraqi patients.

## Patients and methods

This was a prospective, clinical interventional study conducted from September 2016 to December 2018, whereby all patients consulting us with severe (refractory) OAB syndrome were evaluated and those who adhered to our inclusion criteria were consequently included.

Inclusion criteria were: female patients aged >18 years, who presented with long-standing OAB symptoms (dry and wet); refractory to first-line treatments and antimuscarinic drugs (at least two types of antimuscarinic agents were tried for a period ≥3 months). Only patients with idiopathic OAB were included; with the number of voids per day ≥8 and for those with OAB-wet the number of leakage episodes per day ≥2. Patients were excluded if they had significant BOO or a high post-void residual urine volume (PVR) or stress urinary incontinence (UI).

Patients were assessed by history, physical examination, a 3-day voiding diary, urine analysis, renal function tests, ultrasonography (US) and urodynamics (UDS), and all the included patients signed a consent after they were well informed about both types of treatment (SNM and BTX).

The choice of operation was according to patient preference and sometimes according to availability of equipment in our hospital, so the patients were assigned to one of two groups:

Group 1, patients were treated with cystoscopic intradetrusor injections of BTX; and Group 2, patients were treated with SNM.

In both groups, assessment of the treatment outcome was subjective, by assessment of symptoms, patient satisfaction; using the Treatment Benefit Scale [8] and a modified QoL scale; and objective, by using a voiding diary, urine culture and abdominal US (to assess PVR).

Patients scheduled for the 6-month follow-up were only those who showed a reduction in their symptoms (≥50%) early after BTX injection or during the test phase of SNM.

The primary endpoints were the change from baseline in the number of leakage episodes per day, number of voids per day, and the number of patients reporting positive response to treatment at 6 months. Other endpoints included: QoL, patient compliance with each treatment method, complications, and cost of each method.

### Modified QoL scale

After studying many QoL scales, we designed a simplified scale, which was more suitable for our group of patients (according to their educational and cultural characteristics). This scale evaluated three parameters: (i) social embarrassment, (ii) sexual impact, and (iii) psychological impact, and it consisted of three grades for each parameter: 1 = too much, 2 = moderately, 3 = not at all.

Our simple tool to verify patient compliance with the type of treatment was by asking the patient one question: ‘Do you feel comfortable and want to continue with this type of treatment?’, with an answer of ‘yes’ or ‘no’.

### Operative techniques

#### SNM

This type of treatment is accomplished by a two-stage procedure:

**First stage (test phase**): Under general anaesthesia (without muscle relaxant), the patient is placed prone, and with the aid of fluoroscopy a percutaneous tined-lead electrode is placed through the sacral foramen and positioned near the S3 nerve root. This lead is connected to an external pulse generator.

During the test period, which usually last for 2–4 weeks, if SNM does not achieve an improvement in urinary symptoms (even with changing the programme), the lead electrode is removed, but if symptoms improve (i.e. ≥50% reduction) we proceed to the second stage.

**Second stage (permanent phase)**: under general or local anaesthesia, the already placed percutaneous lead is connected to the implantable pulse generator (IPG), which is positioned in the subcutaneous tissue of the buttock. Both stages of the procedure are performed under strict aseptic conditions.

### Intradetrusor BTX injections

Under spinal or general anaesthesia, the patient was positioned in lithotomy, and with the use of a 19.8-F cystoscope, a special injection needle is introduced to the bladder for the injection of the Onabotulinum toxin A (BTX).

For each patient two vials of BTX were used, each contain 100 IU, so in total 200 IU was diluted in 20 mL normal saline, to prepare 20 injections, each of 1 mL (10 IU). The 20 sites were injected inside the bladder according to a map involving three rows of supratrigonal sites, in addition to two injections at the midline of the trigone (away from the ureteric orifices).

At the end of the procedure, a 16-F Foley catheter was inserted and kept *in situ* for 3–5 days

This study was approved by the Ethics Committee of our hospital and by the Scientific Council of the Iraqi Board of Urology.

Analysis of data was carried out using the Statistical Package for the Social Sciences (SPSS ®), version 25 (SPSS Inc., IBM Corp., Armonk, NY, USA). Statistical significance was considered at *P* < 0.05.

## Results

During the study period, 37 patients adhering to our inclusion criteria were assigned to one of the two treatment groups; 18 patients in Group 1 (BTX injections) and 19 in Group 2 (SNM).

In the SNM group, four patients did not achieve the minimum 50% reduction in symptoms in the test phase, so they were excluded from the study. While the other 15 patients (the positive responders) proceeded to the permanent IPG implant phase and were included in the follow-up schedule.

In the BTX group, two patients did not respond well early after the injections and they were excluded, while 16 patients responded well (≥50% reduction in symptoms) and thus they were included in the follow-up schedule.

The demographic features of the included patients were comparable in both groups as shown in Table 1, with a mean age of 43.8 vs 37.2 years in the BTX and SNM groups, respectively.

**Table 1.** Demographic data of the patients.

Variable	BTX group	SNM group	*P*
Age, years			0.468
<30, *n* (%)	3 (18.8)	4 (26.7)	
30–39, *n* (%)	3 (18.8)	4 (26.7)	
40–49, *n* (%)	4 (25.0)	5 (33.3)	
50–59, *n* (%)	3 (18.8)	2 (13.3)	
≥60, *n* (%)	3 (18.8)	–	
Mean (SD, range)	43.8 (14.1, 20–66)	37.2 (11.1, 20–58)	
Marital status, *n* (%)			0.809
Married	10 (62.5)	10 (66.7)	
Not married	6 (37.5)	5 (33.3)	
Occupation			0.832
Worker	7 (43.8)	5 (33.3)	
Housewife	7 (43.8)	8 (53.3)	
Student	2 (12.5)	2 (13.3)	

Regarding the pre-treatment data, OAB was of the dry type in five patients and wet in 11 in the BTX group; and dry in five patients and wet in 10 in the SNM group. Detrusor overactivity was diagnosed by UDS in all the patients. Voiding diary variables were comparable in both groups without a statistically significant difference, as shown in Table 2.

**Table 2.** Voiding diary variables before and after treatment.

Variable	BTX group	SNM group	*P*
Number of voids per day, mean (SD, range)			
Before treatment	16.1 (3.0, 11–22)	14.9 (3.0, 10–22)	0.283
After treatment	8.1 (3.1, 5–18)	6.7 (2.3, 4–12)	0.173
*P*	<0.001*	<0.001*	
Voided volume, mL, mean (SD, range)			
Before treatment	90.8 ± 18.7 (65–130)	87.5 ± 18.5 (60–125)	0.627
After treatment	211.3 ± 72.1 (80–350)	243.7 ± 84.4 (100–400)	0.259
*P*	<0.001*	<0.001*	
Leakage episodes per day, *n*, mean (SD, range)			
Before treatment	3.6 ± 3.1 (0–9)	3.7 ± 3.3 (0–9)	0.971
After treatment	0.8 ± 1.8 (0–7)	1.1 ± 2.0 (0–6)	0.711
*P*	<0.001*	0.002*	

*Significant difference between independent means using Student’s *t-*test or two dependent means using paired *t*-test.

The pre-treatment results of the modified QoL scale were also comparable between the groups and for all the studied parameters (social, sexual and psychological impacts), without a statistically significant difference (Table 3).

**Table 3.** Impacts on QoL variables before and after treatment.

		BTX group		SNM group		*P*	
Effect on		Before	After	Before	After	Before	After
Social activity, *n* (%)	Too much	10 (62.5)	2 (12.5)	11 (73.3)	–	0.519	0.365
	Moderate	6 (37.5)	3 (18.8)	4 (26.7)	3 (20.0)		
	Not at all	–	11 (68.8)	–	12 (80.0)		
	*P*	<0.001*		<0.001*			
Sexual activity, *n* (%)	Too much	8 (50.0)	1 (6.3)	8 (53.3)	3 (20.0)	0.971	0.065
	Moderate	2 (12.5)	–	2 (13.3)	3 (20.0)		
	Not at all	6 (37.5)	15 (93.8)	5 (33.3)	9 (60.0)		
	*P*	0.004*		0.164			
Psychological status, *n* (%)	Too much	11 (68.8)	2 (12.5)	10 (66.7)	3 (20.0)	0.901	0.667
	Moderate	5 (31.3)	10 (62.5)	5 (33.3)	7 (46.7)		
	Not at all	–	4 (25.0)	–	5 (33.3)		
	*P*	0.003*		0.011*			

*Significant difference between proportions using Pearson chi-square test at 0.05 level.

Postoperatively, and throughout the follow-up of the positive responders, we recorded a diminution in response in two patients in the BTX group and three in the SNM group (Table 4). Nevertheless, the mean of all voiding diary variables (at 6 months) remained significantly better than the pre-treatment levels in both groups, but there was no statistically significant difference in the final postoperative levels between the two groups (Table 2).

**Table 4.** Treatment benefit scale and compliance with treatment at 6-months follow-up.

		BTX group	SNM group	
		*N* (%)	*N* (%)	*P*
Treatment benefit scale	Greatly improved	7 (43.8)	7 (46.7)	0.778
	Improved	7 (43.8)	5 (33.3)	
	Not changed	2 (12.5)	3 (20.0)	
	Worsened	–	–	
Treatment benefit scale	Positive response	14 (87.5)	12 (80.0)	0.570
	Negative response	2 (12.5)	3 (20.0)	
Compliance with treatment (feel comfortable)	Yes	10 (62.5)	8 (53.3)	0.605
	No	6 (37.5)	7 (46.7)	

All the responding patients had an improvement in their overall QoL after both types of treatment and it was a statistically significant difference from the pre-treatment levels for all the parameters studied, apart from impact on sexual performance, which was not significantly improved in the SNM group, and again there was no significant difference in the final postoperative QoL parameters between the two groups (Table 3).

The final evaluation at 6 months after treatment revealed that 10/16 and eight of 15 patients felt comfortable with their treatment method, according to the patient compliance scale, in the BTX and SNM groups, respectively (Table 4).

Complications and adverse events of treatment are listed in Table 5, with an obvious difference in the type of complications between the two groups in accordance with differences in the mode of delivery and mechanism of action of each type of treatment.

**Table 5.** Complications in both groups.

Complications (adverse events)		BTX group	SNM group	*P*
UTI (culture and sensitivity positive), *n* (%)	Yes	4 (25.0)	2 (13.3)	0.411
	No	12 (75.0)	13 (86.7)	
PVR, mL, mean (SD, range)		51.9 (72.4, 0–250)	16.3 (17.3, 0–50)	0.075
Need for CIC, *n* (%)	Yes	2 (12.5)	–	–
	No	14 (87.5)	–	–
Diminution in response, *n* (%)	Yes	2 (12.5)	3 (20.0)	0.570
	No	14 (87.5)	12 (80.0)	
Pain, *n* (%)	Yes	2 (12.5)	4 (26.7)	0.318
	No	14 (57.5)	11 (73.3)	
Implant infection, *n* (%)	Yes	–	3(20.0)	–
	No	–	12 (80.0)	
Dislodgment, *n* (%)	Yes	–	2 (13.3)	–
	No	–	13 (86.7)	

CIC, clean intermittent catheterisation.

In our setting, the treatment was much more expensive in the SNM group (cost of the InterStim set, two operations and more follow-up visits).

## Discussion

Interesting findings in our patients were the severe bothering symptoms at presentation (both for OAB wet and dry) as shown in Table 2 and the younger age group in comparison with other studies [9–11]. This may be explained by the fact that younger patients are usually more sensitive about such symptoms having a more negative impact on their QoL, especially for the married and working ladies.

UDS were performed in all of our present patients as an important part of the preoperative evaluation. In fact, UDS are not only used to document detrusor overactivity, but are also helpful to guide the choice of second-line treatment options, e.g. patients with poor detrusor contractions during the voiding phase, high PVR and a low flow rate are not good candidates for BTX because they are more liable to develop urinary retention. Moreover, UDS are also helpful to diagnose other causes of UI (especially stress UI) [12].

The early response of our patients to both types of treatment was good, with statistically significant improvements in all the voiding diary variables, which is comparable to other studies [10,11,13].

Three patients in the SNM group had a diminution in response during the follow-up months, which was either due to IPG dysfunction or explained by the theory that possibly different brain areas were affected during different periods of neuromodulation (early and late), which may account for the change in response in some patients with time [7]. Weil et al. [14] also reported an important association between late loss of therapeutic response and the existence of former psychiatric problems. In the BTX group, two patients reported a gradual diminution in response at 3 and 4 months postoperatively; both were severely depressed (since the preoperative evaluation) and both were from the older age group in our present series.

Despite the obvious improvement in postoperative QoL scores in both groups, when looking in detail, we noticed an interesting change in the psychological impact parameter, in that, preoperatively patients were anxious or depressed because of the fear of wetting, the necessity of frequently going to the toilet, with all the related embarrassments; while postoperatively, when most of these bothering symptoms improved, other types of fear/stress emerged, e.g. in the BTX group, many patients were worried about the possibility of having urinary retention, especially in those with the higher PVRs, also they were anxious and worried as they knew that the effect of BTX was temporary. In the SNM group, many patients were anxious because of the need for frequent visits for strict follow-up and re-programming of the IPG (to maintain the patient on the best response possible parameters). They also considered the device as a foreign body and with its lead and connecting wires restrictive of their daily movements, practicing sports and sexual performance, and it was even felt as a stigma in some young patients (especially the unmarried with low educational level).

All the above mentioned factors were also the reason for the poor compliance with either type of therapy in some of our patients (six of 16 and seven of 15 in the BTX and SNM groups, respectively).

The percentage of those patients who continued to have a positive response at 6 months after intervention was good and comparable in both arms of the study, as has been found in other studies of BTX and SNM [10,15,16]. It was even higher than the percentage of durable response achieved in our earlier study on SNM [7], which is due to the fact that in the earlier study, patients with neurogenic OAB were also included, while in the present study only patients with idiopathic OAB were included.

The complications recorded for both interventions, although different, were relatively few and manageable, which reflects the reasonable safety of both treatment types.

This good safety profile of both types of treatment has been reported in other studies [10,11,15]. Some studies reported higher rates of adverse events with SNM with longer follow-up, with the need for surgical revisions or even permanent removal of the device [16].

Postoperative PVR and UTIs were relatively higher in the BTX group than the SNM group, but only two patients from the BTX group required temporary clean intermittent catheterisation. Thus, the use of Onabotulinum toxin A at a dose of 200 IU (although higher than the traditional 100 IU dose) did not increase the risk of urinary retention or cause any systemic adverse events (e.g. skeletal muscle weakness) and may have played a role in the good response rate achieved. Moreover, our technique of injecting BTX at the trigone, although controversial, was safe and logical for the relief of symptoms by decreasing the irritation in the most sensitive part of bladder, which is the trigone, without leading to VUR.

For those patients with OAB who require a second-line therapy/intervention, there is still insufficient evidence to decide which is better, or what option to start with, BTX or SNM, with disparity in the guidelines of different countries [17,18].

Apart from the clinical indications, one of the determinants for choosing a treatment option is the availability and cost of a technique. In Iraq, SNM (the Interstim device) is not available everywhere, it is more expensive than BTX injections and a very limited number of urologists have the experience in performing this technique, but on the other hand SNM has a wider range of indications than BTX and its therapeutic effect is more durable. In studies conducted in other countries with different healthcare systems, both treatments have a comparable long-term cost effectiveness, and in some instances SNM was more cost effective when its therapeutic effect exceeded 5 years [16,19,20].

The limitations of the present study are the small sample size (as it is a single centre experience), and the lack of randomisation, although all the included patients were eligible for both procedures.

## Conclusions

Both intradetrusor BTX injections and SNM, in our experience, were generally comparably safe and effective in alleviating the bothersome symptoms of our Iraqi patients with refractory OAB during the early postoperative period and after 6 months of follow-up, which was reflected in an improvement in their QoL.

The use of 200 IU BTX, and the intra-trigonal injections technique adopted in the present study, was safe and most likely beneficial.

To choose one of these techniques, many factors should be considered, including UDS findings, the availability of the equipment, the cost of treatment, the skills and experience of the surgeon, and the patients’ expectations, understanding and cooperation.
